# Understanding the relationship between oral health and psychosis: qualitative analysis

**DOI:** 10.1192/bjo.2023.33

**Published:** 2023-04-11

**Authors:** Elizabeth Turner, Katherine Berry, Leah Quinlivan, David Shiers, Vishal Aggarwal, Jasper Palmier-Claus

**Affiliations:** Division of Psychology & Mental Health, University of Manchester, UK; Greater Manchester Mental Health NHS Foundation Trust, UK; and Manchester Academic Health Science Centre, University of Manchester, UK; Division of Psychology & Mental Health, University of Manchester, UK; Manchester Academic Health Science Centre, University of Manchester, UK; and National Institute for Health and Care Research (NIHR) Greater Manchester Patient Safety Translational Research Centre, University of Manchester, UK; Division of Psychology & Mental Health, University of Manchester, UK; School of Dentistry, Faculty of Medicine and Health, University of Leeds, UK; The Spectrum Centre for Mental Health Research, Faculty of Health & Medicine, Lancaster University, UK; and Lancashire & South Cumbria NHS Foundation Trust, UK

**Keywords:** Psychosis, schizophrenia, dental, teeth, qualitative research

## Abstract

**Background:**

Individuals with psychosis have poor oral health compared with the general population. The interaction between oral health and psychosis is likely to be complex and have important ramifications for improving dental and mental health outcomes. However, this relationship is poorly understood and rarely studied using qualitative methods.

**Aims:**

To explore patient perspectives on the relationship between oral health and psychosis.

**Method:**

The authors recruited 19 people with experiences of psychosis from community mental health teams, early intervention in psychosis services, and rehabilitation units. Participants completed a qualitative interview. Transcripts were analysed with reflexive thematic analysis.

**Results:**

The analysis resulted in three themes: theme 1, psychosis creates barriers to good oral health, including a detachment from reality, the threat of unusual experiences and increased use of substances; theme 2, the effects of poor oral health in psychosis, with ramifications for self-identify and social relationships; and theme 3, systems for psychosis influence oral health, with central roles for formal and informal support networks.

**Conclusions:**

Psychosis was perceived to affect adherence to oral health self-care behaviours and overall oral health. Poor oral health negatively affected self-identity and social relationships. Clinical implications include a systemic approach to provide early intervention and prevention of the sequelae of dental disease, which lead to tooth loss and impaired oral function and aesthetics, which in turn affect mental health. Participants felt that mental health services play an important role in supporting people with oral health.

Oral health is important for day-to-day functioning and plays a critical role in eating, smiling and talking.^1^ Conversely, poor oral health is detrimental to general health, self-esteem and quality of life.^2^ People diagnosed with psychotic disorders experience worse oral health outcomes than the general population, including higher levels of decayed and missing teeth.^3,4^ Psychotic disorders (e.g. schizophrenia, schizoaffective disorder) have a lifetime prevalence of 3%^5^ and are characterised by experiences of hallucinations, delusions and difficulties concentrating or thinking.^6^ Psychosis commonly develops between late adolescence and early adulthood,^7^ which is important because research indicates that oral hygiene habits often decline in adolescence.^8^ The prevalence of edentulousness (complete loss of teeth) and periodontal disease are also significantly higher in people with experiences of psychosis.^4,9,10^ Conversely, rates of filled teeth are significantly lower,^11^ which may indicate reduced access to dental care and the need for more intrusive treatments, like teeth extraction. This increases demand in emergency dental treatment and places a significant financial burden on the healthcare system.^12^

Reasons for suboptimal oral health in people experiencing psychosis are likely complex and multifactorial. They may include individual influences, such as motivation to seek treatment,^13^ chronicity of mental health problems^14^ and high-risk behaviours^15^ such as smoking. The prevalence of smoking in people with psychosis appears to be higher than the general population,^16^ and is a known factor in elevating the risk of all oral health problems in the general population.^17^ Iatrogenic effects of psychiatric treatments such as medication may also play a role in the oral health disparity in people with psychosis.^18^

The cost of dental services possibly acts as a barrier for people with experiences of psychosis. In the UK, exemptions may apply for low-income patients. However, the payment system is complex and may be hard for patients to navigate, which may reduce access.^19^ To ensure success of advanced care and protect limited National Health Service (NHS) resources, UK dentists expect patients to have healthy gums and no underlying risk factors like high frequency of sugar intake, smoking or poor oral health self-care behaviours (e.g. regular flossing and tooth brushing).^19^ However, the uptake of oral health self-care behaviours appear low in people with psychosis.^20^ Therefore, this population may find it difficult to engage in key preventative behaviours that could not only reduce the need for emergency treatments,^21^ but also allow a restoration of function and aesthetics.

## Study aims

The aim of this qualitative study was to interview people with psychosis and seek their perspective about the ways in which oral health and psychosis interact. By doing this, we sought to understand the complex relationship and the unique challenges this population face, to address unmet oral health needs. We aimed to capture the important voice of patients to explore what meaningful changes are needed to improve dental outcomes in people with psychosis. To our knowledge, this is the first qualitative study to explore the relationship between oral health and psychosis in a patient sample.

## Method

### Sample and recruitment

A pragmatic qualitative approach^22^ was utilised to ascertain knowledge about the relationship between psychosis and oral health, using semi-structured interviews. We aimed to recruit a sample large enough to develop a rich understanding of the research question.^23^ Clinical teams from two NHS Trusts in England referred patients meeting criteria for an ICD-10 or DSM-IV diagnosis of a psychotic disorder (e.g. schizophrenia, schizoaffective disorder), as diagnosed by the referring clinical service. We also include participants with first-episode psychosis as indicated by meeting the operational criteria for an Early Intervention for Psychosis Service. See Table 1 for full inclusion criteria.

**Table 1:** Criteria for inclusion

Inclusion criteria	Exclusion criteria
18 years or over	Diagnosis of a known moderate to severe intellectual disability or organic disorder (e.g. dementia, acquired brain injury)
Under the care of an out-patient or in-patient team	Currently in crisis with known substantial risks to self/others
A diagnosis of schizophrenia, schizoaffective disorder, delusional disorder, schizophreniform or first-episode psychosis (meeting criteria for entry into an Early Intervention Service)	
Able to give informed consent	
Able to speak English	

### Ethics statement

The authors assert that all procedures contributing to this work comply with the ethical standards of the relevant national and institutional committees on human experimentation and with the Helsinki Declaration of 1975, as revised in 2008. All procedures involving human participants were approved by the North-West – Greater Manchester East Research Ethics Committee (approval number 19/NW/0723). All participants provided informed consent to take part.

### Data collection

The first author (E.T.) conducted face-to-face (*n* = 3) or telephone (*n* = 16; during the COVID-19 pandemic) interviews, following a topic guide developed with input from three people with lived experience of psychosis and one carer. Interviews ranged from 18 to 62 min (median: 31.22, interquartile range: 28–40) and were transcribed verbatim. Participants provided key demographic and clinical information before interview and completed the Oral Health Impact Profile-14 (OHIP-14)^1^ and the Oral Health Survey^24^ to assess their oral health-related quality of life and knowledge of dental risk factors. The majority of the sample were recruited from a community mental health team. Therefore, the majority had chronic mental health difficulties with multiple in-patient admissions.

### Analysis

The analysis process was both systematic and iterative, following guidance for reflexive thematic analysis outlined by Braun and Clarke.^25,26^ The lead author (E.T.) actively engaged with the data-set for familiarisation and completed line-by-line coding, which led to the development of preliminary themes. Authors L.Q. and V.A. coded a subsection of transcripts to provide other perspectives, and the research team developed and refined these themes during regular multidisciplinary discussions. Data was managed with NVivo 12 for Windows (QSR International Ply Limited, Denver, USA; https://www.qsrinternational.com/nvivo-qualitative-data-analysis-software/home).

### Reflexivity statement

The first author (E.T.) was a trainee clinical psychologist with a Master's in Health Psychology and experience of clinical working with psychosis. E.T. engaged with the relevant research on dental literature before analysis, which informed deductive coding.^27^ The data was collected through a psychological lens, given the first author's professional training. During the process of the initial interviews, the first author noted a tendency to communicate approval in the presence of good oral health routines, which may have made it difficult to disclose any difficulties faced. Given the potential for self-stigma and societal beliefs about good oral hygiene, efforts were taken to ensure that interview questions were not asked from a place of judgement, rather to explore individual perspectives on oral health and psychosis. The interviewer explained that she had no professional experience in dentistry and therefore was not adopting a critical stance when exploring oral hygiene and habits.

The research group comprised of two clinical psychologists (K.B. and J.P.-C.), a non-clinical qualitative researcher (L.Q.), a dentist (V.A.) and a carer representative (D.S.). This diversity in perspectives supported a broader interpretation of the data.

## Results

Demographic and dentist visit information is reported in Table 2, alongside results from the OHIP-14 (Supplementary Fig. 1 available at https://doi.org/10.1192/bjo.2023.33). Findings from the Oral Health Survey indicated variable knowledge of oral health related risk factors, compared with a similar aged general population sample.^24^ Study participants demonstrated a slightly better knowledge of risk factors pertaining to erosion (47% correct response rate compared with 31%) and caries (51% and 47%, respectively) than the general population sample. However, risk factor knowledge of periodontal disease was greatly reduced, with the study sample answering 30% of questions correctly, whereas 77% of the general population sample provided a correct response. Table 2 shows the percentage of participants experiencing difficulties in each oral health-related quality of life domain of the OHIP-14. A high proportion of the sample reported difficulties in all domains of the OHIP-14.

**Table 2:** Demographic information and dental data (*n* = 19)

Age, years	
Median	44
Interquartile range	33–52
Male, *n* (%)	12 (63%)
Ethnicity, *n* (%)	
White British	15 (80%)
Black British	1 (5%)
Asian British	1 (5%)
White other	1 (5%)
White Irish	1 (5%)
Service, *n* (%)	
Community Mental Health Team	16 (84%)
Early Intervention in Psychosis Service	1 (5%)
Rehabilitation unit	2 (11%)
Diagnosis, *n* (%)	
Schizophrenia	16 (84%)
Schizoaffective disorder	3 (16%)
Number of in-patient admissions	
Median	3
Interquartile range	7 (2–10)
Other diagnoses, *n* (%)	
Depression	6 (31%)
Anxiety disorder	3 (16%)
Bipolar disorder	1 (5%)
Obsessive–compulsive disorder	1 (5%)
Post-traumatic stress disorder	1 (5%)
Dissociative identity disorder	1 (5%)
Eating disorder	1 (5%)
Autism spectrum disorder	1 (5%)
Physical health difficulties, *n* (%)	
Back problems	2 (11%)
Diabetes	1 (5%)
Asthma	1 (5%)
Mobility problems	1 (5%)
Chronic obstructive pulmonary disease	1 (5%)
Arthritis	2 (11%)
Psoriatic arthritis	1 (5%)
Obesity	1 (5%)
High blood pressure	1 (5%)
High cholesterol	1 (5%)
Graves' disease	1 (5%)
Sydenham's chorea	1 (5%)
Antiphospholipid syndrome	1 (5%)
Registered with a dentist, *n* (%)	
Yes	16 (84%)
Last dental contact, *n* (%)	
<6 months	12 (63%)
<12 months	6 (31%)
<5 years	1 (5%)
Oral Health Impact Profile-14 results (*n* = 18)	
Physical pain	94%
Psychological discomfort	89%
Psychological disability	78%
Functional limitation	42%
Physical disability	53%
Social disability	56%
Handicap	72%

### Theme 1: psychosis creates barriers to good oral health

Table 3 includes further supporting quotes from the data. The first theme related to participants’ perception of the ways in which psychosis directly and indirectly affected oral health. Many participants reported feeling like poor oral health was an inevitable consequence of psychosis despite attempts to maintain a healthy routine.

**Table 3:** Additional supporting extracts for all themes

Theme	Extract	Source
Theme 1: psychosis creates barriers to good oral health		
A psychotic disconnect from self-care	‘Erm, couple of times, like I haven't brushed my teeth, like if I've been a bit poorly like, ‘cos you don't really look after yourself do you, ‘cos you don't, you're not really with it. When people are under stress which could be considered psychosis, it's like they've got too much on their mind to brush their teeth., I'd say it's like brushing your teeth seems to be kind of how do you say it, less a priority.’	Participant 16
	‘I'm not really there to be able to look after myself as well.’	Participant 10
	‘It's harder when you've got psychosis ‘cos sometimes you don't know time of day kind of thing, you just try and do what you can to get through it.’	Participant 12
The threat of first-rank psychosis to oral health	‘…They were related they were connected like the voice would play mind games with my tooth pain. So, it would be, they would be connected.’	Participant 6
	‘I felt like I was being talked about at the dentist. I felt like they were saying she stares or whatever and I didn't feel comfortable going in there, I felt like they were all talking about me in there.’ ‘Yeah I do get a bit sort of anxious you know with, in the waiting room because my voices start to flare up in that situation. I think I sort of get in my head that people can hear my voices, which is making me uncomfortable.’	Participant 9 Participant 7
	‘So it was interesting I had a friend who had this conspiracy theory of that the royal family put like a fluoride or something in the toothpaste to stop you, to prevent you from becoming awake and conscious and stuff and that played a bit on my mind. So as soon as then I used to take a minimum but like I say I'd do less because of that that but it's just paranoia.’	Participant 6
Chemical inevitability	‘I won't think twice about taking drugs and drinking alcohol because of my psychosis. Because of the way my psychosis, my general mood and well-being can sometimes be a bit low and a bit erm unstable that will drive me to drink alcohol and take drugs, So that won't help my mouth, my, that definitely doesn't help my teeth and mouth, you know.’	Participant 21
	‘I started smoking it's something you know it's a lot of percentage of mentally ill people who smoke. I don't know the statistics. It's just something you think calms your nerves or whatever. Make me lose my teeth it was terrible and I didn't want, I didn't want to. It's against my will to smoke.’	Participant 6
	‘I was sort of like, drinking a lot and you know I did go through a little bit of stage, of a stage taking a few drugs and whatnot, erm, so, I presumed that it was something to do with that really. ‘Cos if you think about it, if you're taking drugs and that, you know, and you're up until like 5 and 6 o'clock in morning, you're not brushing your teeth before you go to sleep.’	Participant 13
	‘So when you're having a bad day with your psychosis and you want to escape it, you start taking smack, your teeth mouth and gums are affected.’	Participant 18
	‘Well the thing is, this medication knocks me out, at night-time I just go to sleep so I don't like, I want to go to sleep.’	Participant 9
Theme 2: the impact of poor oral health in psychosis		
Oral health and self-identity	‘Erm, it's ok, like it used to be one of the things that I only liked, like the only thing that I liked about myself, but I guess it's a bit different now, ‘cos like they're not as good my teeth, but I just try and do what I can.’	Participant 10
	‘I was quite upset when that happened, you know, even though the dentist said oh it looks great and everything. I suppose, you know, I suppose when it gets to that stage, you know, I, it did affect me quite a lot, but now, I'm quite used to it, so I've just accepted it really.’	Participant 13
	‘It made me feel like there was no point in me looking after myself because I was dirty anyway and there was no point in looking after myself because I couldn't make myself any better, any cleaner.’	Participant 20
	‘My gosh, they (the voices) don't half, put you down, you know, they're constantly at you all the time, they, you know, when me teeth were broken, they were constantly at me.’	Participant 11
Oral health and relationships with others	‘I felt dirty. I wanted to feel very dirty because. I didn't wanna be attractive anymore. I didn't wanna be attracted by men, you know, it was a bit deeper than that so it was, yeah, I wanted to be unattractive.’	Participant 11
	‘..Well basically, I knew I had bad breath at the time, so when I was talking to someone, I would talk under my breath instead of breathing out fully.’	Participant 20
	‘I mean nowadays I probably smile less because my teeth are yellow.’	Participant 21
Theme 3: systems for people with psychosis influence oral health		
The impact of system-level support	‘I've got my community mental health team and they help me but I wouldn't ask them something that's totally out of reach.’	Participant 18
	‘It might have made a bit of difference. It might not have got out of hand if I'd had support with it. Cos it's not really talked about.’	Participant 8
	‘Well I've spoken to my support worker and he's told me he's gonna try get me into a dentist. I'm gonna have to ask [care coordinator] as well to sort of get the ball rolling.’	Participant 19
Psychosis and informal support for oral health	‘My sister, my sister is my carer. She will always tell me to brush twice and use my mouthwash.’	Participant 21
	‘I think so yeah, you've got somebody next to you, you know if you need to talk you know but nothing specific but just somebody to sort of keep you calm before you go in.’	Participant 2

#### A psychotic disconnect from self-care

This subtheme refers to the all-encompassing nature of a psychotic episode that led to a disconnect with everyday activities, including oral health care. Participants talked about how psychosis brought about a temporary detached or dissociative state, which meant that self-care, like tooth brushing, felt distant and unobtainable. There was a complete preoccupation with their psychotic experiences, which made it impossible to maintain an oral health routine.

‘Yeah, it's like listening to the voices and the hallucinations and you're wrapped up in that world. That's a complete delusional fantasy and you're not in the real world to brush your teeth.’ Participant 19.

These feelings of disconnect from reality resulted in an inability to attend to oral health self-care behaviours (e.g. tooth brushing, flossing, dental visiting), which could trigger periods of overcompensating by overbrushing once acute psychosis subsided. Fluctuating patterns of behaviour suggested that people had an intrinsic sense of the importance of maintaining oral health, but that this was disrupted during periods of psychosis.

‘There's two sides to it really. You can either be, sort of, like obsessed about it and try and keep your mouth and your teeth as clean as possible, or when you're in a psychosis where time just goes without you realising, you know, hours and hours might pass, and you're not brushing your teeth.’ Participant 13.

#### The threat of first-rank psychosis to oral health

Participants talked about the direct impact that hallucinations and unusual beliefs had on their oral health, often operating through fear and anxiety. Such experiences prevented people taking care of their mouth, teeth and gums, and made them worried about accessing treatment. For example, threatening command hallucinations sometimes directly warned of possible negative consequences if a person engaged in dental treatments, which affected future dental attendance.

‘One of the voices said if you take your wisdom tooth out, we're gonna bring more voices in your head and then they took my wisdom tooth out and three more voices came.’ Participant 1.

Conversely, psychotic beliefs did not always impact adversely on dental health care. One participant expressed that their self-reported unusual beliefs, which appeared to be more grandiose in nature, enhanced their visits to the dentist. This suggests that it was also possible for psychotic symptoms to have a positive impact on oral health and potentially increase the likelihood of visiting the dentist.

‘I think because I'm important, they [dental staff] like seeing me and they're very polite to me and they like seeing me ‘cos I'm a bit of a VIP.’ Participant 15.

#### Chemical inevitability

This subtheme concerns the perceived impact that illicit and non-illicit substances have on oral health. People considered side-effects from antipsychotic medication to be damaging to oral health, and described finding it difficult to maintain a regular oral health routine because of the side-effects of tiredness and fatigue, with night-time routines being particularly challenging. Direct damage was thought to be caused by other side-effects, including dry mouth and cravings for sugary food. There was also some recognition that antipsychotic medication could positively affect oral health routines by minimising preoccupation with psychotic experiences.

‘I think it's a really bad side effect. You need saliva and that to keep your teeth in good order, good working order sort of thing, it's a really bad side effect.’ Participant 8.

The loss of routine and perceived direct damage from medication was mirrored by the perceived effects of other substances. This included illegal drugs, alcohol, caffeine and tobacco. Participants described these substances going ‘hand in hand’ (participant 18) with psychosis. They attributed their use of substances to escapism and distress management. There was some overlap with the subtheme ‘a psychotic disconnect from self-care’, as participants voiced a detachment from the real world when intoxicated, which affected their ability to attend to oral health self-care behaviours. Typically, participants recognised that substances negatively affected their oral health either by hindering their routine or directly causing damage. Despite this, participants stated that using illegal substances had often taken priority over self-care, because of the power of addiction and beliefs about substances reducing distress.

‘To be honest it was probably the same time when I was smoking crack, so probably drugs over dentist.’ Participant 4.

### Theme 2: the impact of poor oral health in psychosis

The second theme relates to the ways in which participants’ thought that their oral health affected self-identity and their ability and desire to connect with others. The theme encompasses the impact that poor oral health has on psychological constructs that are central to mental health, such as self-esteem and sense of identity. Participants felt that social relationships were also affected by dental deterioration. Participants conceptualised the interplay between oral health and mental health in a destructive cycle. Poor mental health led to a worsening state of oral health that, in turn, further affected mental health.

#### Oral health and self-identity

This subtheme refers to the ways in which oral health affects peoples’ sense of self and identity, in the context of psychosis. Participants believed that the appearance of their mouth, teeth and gums were fundamental to their value as a person. Those who had experienced dental deterioration commonly described themselves as ‘dirty’. Existing concerns about the appearance of the oral cavity sometimes manifested as critical auditory hallucinations, which affected self-esteem.

‘The voices might call you a tramp or things like that and you know, threaten to knock out you know the rest of your teeth and things like that.’ Participant 19.

Overall, deterioration in oral health was an emotive topic for participants. The perceived loss of good oral health seemed to lead to a sense of loss in identity. It was as if the deterioration stripped away remaining positive attributes, such as their smile.

‘I've always had a big smile, you know. People used to comment that I had a nice smile, so it was upsetting.’ Participant 8.

#### Oral health and relationships with others

The cumulative impact of psychosis and poor oral health made social and romantic relationships challenging. People expressed feeling vulnerable and in danger from others because of their paranoia, and this was further exacerbated by their poor oral health.

‘It's like I say, when I'm going out and things like that as well because I'm paranoid anyway about going out just in case, I mean if I'm thinking of going out, you know, what if someone sees my teeth and wants to start trouble with me, they think I'm an easy target or something like that.’ Participant 19.

Fears of negative judgement from others about their oral health seemed to perpetuate social avoidance. This related to the subtheme ‘oral health and self-identity’, suggesting that people experienced a reduction in self-esteem because of deteriorating oral health, which affected the way they viewed themselves and how they expected others to perceive them.

To mitigate the effects of poor oral health in social situations, participants attempted to mask their oral health when smiling or talking to others, to avoid embarrassment or shame. This affected their ability to have authentic connections with others. Masking included hiding their teeth when smiling and talking to people under their breath to disguise halitosis (bad breath). Poor oral health was also conceptualised as a strategy to keep people away in the context of experiences of early trauma, suggesting that this can sometimes be an active process to protect the self from further danger.

‘I find it quite difficult to smile because I think me teeth are that bad, even though, no one can tell, erm, I know, you know, so, you know, I, sort of like maybe cover my mouth with my hand or, try and smile without actually opening my mouth.’ Participant 13.

### Theme 3: systems for people with psychosis influence oral health

This theme relates to the ways in which the presence of a psychotic disorder can influence how formal and informal systems interact with oral health. The lack of prioritisation of oral health in some mental health services was juxtaposed with the holistic care provided by others. Informal support from family and friends was a central factor in maintaining good oral health in this population.

#### The impact of system-level support

This subtheme relates to the way in which the presence of a psychotic disorder can influence professional support systems. Participants talked about having support around them because of their psychosis, but that this support was designed to treat their mental health and that there was little consideration of wider physical health needs, including their oral health. In general, mental health professionals seldom asked about patients’ oral health because their psychotic experiences were considered to be the priority. This passivity of healthcare professionals in relation to dental health mirrored patient perceptions that poor oral health was inevitable and may have contributed to a lack of prioritisation of oral health behaviours.

‘So I guess in that sense, like they (mental health professionals) never, I don't think they ever think of that as a, sort of, thing that they need to address, like the mental health team.’ Participant 10.

A neglect of oral health needs was not always the case. Practical support from mental health teams to access the dentist (e.g. making the appointment or providing transportation) sometimes positively affected dental visits. More specialised psychological support to reduce dental fear also enabled access to the dentist by reducing psychological barriers. Support workers, nurses and psychological therapy staff provided oral health support to participants at times, which was warmly received. People who received tailored and holistic support spoke positively of their care team.

‘Me and [healthcare professional's name] done some work on it to like, to think about safe place when I'm in dentist chair and that's worked, that's been really helpful.’ Participant 12.

#### Psychosis and informal support for oral health

Support from family and friends in people with psychosis was instrumental in maintaining oral health. The presence of a key family member or trusted friend made it easier to attend dental appointments and provided support following treatment. Informal support networks advocated for better dental treatment for people with psychosis. However, potential difficulties in connecting with others when oral health was poor (as identified in the ‘oral health and relationships with others’ subtheme) may have affected the availability of this important source of support for some participants.

‘A friend, he's gone he's asked after my visit to do a better denture for me because they've done a bridge but they can't do it.’ Participant 2.

Participants recognised that sometimes intervention from close family or friends was needed, particularly in the form of reminders if self-care was low as a consequence of psychosis or associated difficulties (e.g. substance misuse). However, the heightened focus on tangible difficulties like oral health was sometimes perceived by participants as uncaring and neglectful of emotional needs.

‘Yeah, cos sometimes I need it but sometimes I want her to say what's wrong with you? Are you okay? But sometimes she's just like, you've not brushed your teeth.’ Participant 18.

## Discussion

### Main findings

This is the first qualitative study to explore patient perspectives on the perceived relationship between oral health and psychosis. Our analysis suggests that people with psychosis perceive a relationship between psychosis and oral health, whereby both factors affect each other to some degree. Symptoms and behaviours associated with psychosis inhibit oral health behaviours and cause direct damage to teeth and gums. Poor oral health can then disrupt self-identity and the ability to form relationships with others. The interplay between oral health and psychosis was conceptualised in this paper as a negative feedback loop, whereby poor mental health reduced oral health behaviours, leading to deteriorating oral health, which then negatively affected mental health. The presence of formal and informal support for dental needs was important in breaking this vicious cycle by increasing access to the dentist for dental treatment. In addition, informal networks provided everyday support, which increased the likelihood of participants performing key oral health self-care behaviours.

### Comparison with wider literature

The current study in a small sample of people living with psychosis potentially elucidates the reasons for low uptake of oral health behaviours in people with psychosis.^20^ This includes a sense of inevitability that people felt, resulting from a disconnection from reality, the fear caused by psychotic experiences, and the use of prescribed medication and illicit and non-illicit substances. Past research has suggested that feelings of detachment can negatively affect general functioning,^28^ and the present study indicates that this may extend to oral healthcare. Our analysis also suggests that fear and anxiety caused by hallucinations and delusions may prevent people from engaging in oral hygiene practices, adding to the burden of these experiences. In the absence of active psychotic symptoms, participants reported finding it difficult to attend the dentist and perform oral health behaviours. Therefore, support may be required even when the individual is mentally more able to prioritise oral health.

Participants perceived substances to be closely associated with psychosis and recognised that these were contributors to poor oral health, which is consistent with the wider literature.^29^ This is consistent with research indicating that lifestyle factors such as smoking can double the risk of periodontal disease.^15^ A recent review suggested that antipsychotic medication affects functioning because it causes feelings of lethargy and reduced motivation,^30^ which could affect self-care behaviours like tooth brushing. The present findings also highlight the impact of other medication side-effects such as xerostomia (dry mouth) and an increased desire for sugary foods, which are risk factors for dental caries.^24^ A recent qualitative study also suggested parallels between the use of substances (illicit and non-illicit) and antipsychotic medication and its effect on oral health.^31^

Recent research has suggested that many people with psychosis experience appearance-related voice content, particularly around weight gain which can negatively affect self-esteem and lead to social withdrawal.^32,33^ This is supported by our data, as participants disclosed auditory hallucinations that focused on the health of their mouth, teeth and gums, and often contributed to social distancing.

The current study found formal and informal support systems to be an integral factor in maintaining oral health. Previous findings have indicated that carers often bridge the gaps in support for patients with psychosis.^34^ Our analysis points to the value of informal support networks for people with psychosis and comorbid with dental health needs. However, some participants did not have access to this type of support. The data also showed that the level of support provided by mental health teams is inconsistent. Some services did not routinely prioritise needs that were not within the mental health remit. A move toward more holistic care in mental health services has sometimes been endorsed,^2,35^ and advances have been made to incorporate physical health needs into mental healthcare.^36^

### Strengths and limitations

In terms of strengths, the study involved exploring perspectives in people with lived experience of psychosis, which provides personal context to the issue of psychosis and oral health. Having an interdisciplinary team enabled trustworthiness and triangulation of the analysis.^37^ The research had strong patient and public involvement, resulting in a coproduced topic guide that was acceptable to participants and produced a wealth of relevant information. There are also limitations to the research, which include a lack of diversity in a sample that was predominantly White British. The study ran during the UK COVID-19 lockdown period, which restricted recruitment and made it difficult to adhere to the *a priori* sampling frame. Furthermore, only a minority of participants were experiencing early first-episode psychosis, and there is a need to explore this relationship between psychosis and oral health across a broader continuum of recovery. The study did, however, recruit from two NHS Trusts, which widened the geographical spread of the sample.

### Clinical implications

The findings have key implications for mental health services. The analysis suggests that the maintenance of oral health care may be contingent on mental wellness. Professional and informal support systems may be central to supporting patients’ mental health, enabling access to dental treatment and promoting good oral hygiene behaviours through practical and emotional support. Mental health teams can provide valuable support to patients with co-occurring dental and mental health needs. Our analysis indicates that consideration should be given to the iatrogenic effects of antipsychotic medication, which have relevance to oral health. This could include informed and collaborative decision-making about the potential impact of medication side-effects on people's teeth and gums (e.g. xerostomia/dry mouth). Services could support patients to discuss prescribed medication with their dentist, to ensure that relevant advice and guidance is provided by dental professionals to mitigate possible side-effects. It is well-documented that this population commonly engage in high-risk behaviours such as illicit drug, tobacco and alcohol use.^10^ Modifiable behaviours like smoking should be targets for intervention.^15^ However, the current results indicate that these are often used as a form of distress management, and therefore it is prudent that mental health and dental professionals use a non-judgemental approach (e.g. motivational interviewing) to explore the function and any resistance to reducing these behaviours, despite the negative impact on their oral health.

Our analysis suggests a negative feedback loop between oral and mental health. Interventions should target the avoidance of oral health behaviours (e.g. visiting the dentist, tooth brushing, flossing) to break negative and perpetuating cycles of behaviour. A recent consensus statement^38^ has advocated that poor oral health should not be an inevitable consequence of severe mental illness, and has set targets for addressing current inequalities. In line with the present study findings, one of the targets is to ensure that training is provided to relevant professionals (e.g. dental and mental healthcare workers) to facilitate understanding and management of oral health difficulties in people with experiences of psychosis. Our data indicate that practical support from mental health teams and informal networks can help to facilitate access to dental care and treatment (e.g. support to book dental appointments, transportation, prompting patients to bring a supportive friend or family member if this is helpful). Therefore, such practical support must be encouraged to promote timely dental care. Based on the current findings, specific interventions may include formulating the origins of avoidance, utilising motivational strategies to improve adherence to oral health routines, and psychological strategies to reduce dental anxiety. There is also a need for services to foster hope and promote optimism for change.

In conclusion, patients perceived a bidirectional relationship between psychosis and oral health. Psychosis affected people's ability to attend to oral health needs and difficulties with oral health then perpetuated worsening mental health. Mental health services and informal support networks play a key role in supporting oral health needs and facilitating access to dental treatment. People experiencing psychosis often viewed poor oral health as an inevitable consequence of their situation. However, oral health problems are preventable and treatable. Clinical services must do more to break the vicious cycle between oral health and psychosis.

## Data Availability

The data that support the findings of this study are available on request from the corresponding author, E.T. The data are not publicly available due to restrictions to protect the confidentiality of research participants.

## Supplementary material

For supplementary material accompanying this paper visit http://doi.org/10.1192/bjo.2023.33. click here to view supplementary material
