# Supplementary material for: Understanding the relationship between oral health and psychosis: qualitative analysis
Source: BJPsych Open. 2023 Apr 11;9(3):e59. doi: 10.1192/bjo.2023.33 (PMC10134239; doi:10.1192/bjo.2023.33)
Supplement: Supplementary file 1 [file S2056472423000339sup001.docx]

**Supplementary material**

Results from Oral Health Impact Profile-14

Results from Oral Health Survey

| Risk factor knowledge domain | Correct response rate study sample (%) | Correct response rate general population sample (%) |
| --- | --- | --- |
| Caries | 51 | 47 |
| Periodontal disease | 30 | 77 |
| Erosion | 47 | 31 |
